# Right Ventricular Thrombus: A Rare but Potentially Fatal Condition

**DOI:** 10.7759/cureus.93285

**Published:** 2025-09-26

**Authors:** Se Jong Choi, Jason Dagoon, James Park, Chul Chae

**Affiliations:** 1 Internal Medicine, School of Medicine, California University of Science and Medicine, Arrowhead Regional Medical Center, Colton, USA; 2 Radiology, School of Medicine, California University of Science and Medicine, Arrowhead Regional Medical Center, Colton, USA; 3 Radiology, Arrowhead Regional Medical Center, Colton, USA; 4 Medical Imaging, Arrowhead Regional Medical Center, Colton, USA

**Keywords:** echocardiography, hypercoagulable state, obstructive shock, percutaneous thrombectomy, pulmonary embolism (pe), right ventricular thrombus, urothelial malignancy

## Abstract

Right ventricular thrombus (RVT) is a rare but potentially fatal entity, often representing a thrombus in transit with high risk for embolization and obstructive shock. Management is particularly complex in oncology patients, where anticoagulation and thrombolysis may be contraindicated.

We present a 49-year-old woman with newly diagnosed, locally advanced urothelial carcinoma (T4N3M0), who developed a 3.0 × 2.2 cm mobile thrombus within the right ventricular outflow tract (RVOT), detected by transthoracic echocardiography and corroborated by contrast-enhanced chest CT. Her course was complicated by active vaginal bleeding, thrombocytopenia (platelets: 40-60 × 10⁹/L), and coagulopathy (international normalized ratio: 1.6), precluding anticoagulation or systemic thrombolysis. Catheter-directed thrombectomy with AngioVac (AngioDynamics Inc., Latham, NY) was attempted but was unsuccessful. Pathology confirmed a bland thrombus, excluding tumor invasion. Despite placement of an inferior vena cava (IVC) filter, she suffered obstructive shock with hypotension (systolic blood pressure: ~70 mmHg), hypoxemia (oxygen saturation: 70% on supplemental oxygen), and supraventricular tachycardia, requiring cardioversion, vasopressors, and intubation. Her condition further declined with extensive bilateral deep vein thromboses, methicillin-resistant *Staphylococcus aureus* (MRSA) bacteremia requiring intravenous antibiotics, progressive anemia, and metastatic progression despite two cycles of MVAC (methotrexate, vinblastine, doxorubicin, and cisplatin) chemotherapy. Following a multidisciplinary review, curative options were exhausted, and she transitioned to comfort care.

This case illustrates the diagnostic challenges and therapeutic limitations of RVT in cancer patients with contraindications to standard therapies. Prognosis is poor when anticoagulation and thrombolysis are not feasible, and no consensus guidelines exist for this population. Early multidisciplinary coordination and integration of palliative care are essential to balance aggressive interventions with quality-of-life considerations.

## Introduction

Right ventricular thrombus (RVT) is an uncommon but high-risk finding, often occurring in the setting of acute pulmonary embolism (PE), where it may represent a thrombus in transit from the venous circulation [1,2]. Other causes include right ventricular dysfunction, indwelling devices, hypercoagulable states such as malignancy, and procedural complications [3-5].

The estimated prevalence of RVT among patients with PE is 2-4% on echocardiography, with even lower rates outside the context of PE [6,7]. RVT is associated with right ventricular dilation, dysfunction, and poor prognosis, including risk of embolization, hemodynamic collapse, or sudden death [5,7].

Diagnosis is most often achieved by transthoracic echocardiography (TTE), which can identify mobile or free-floating thrombi. Transesophageal echocardiography (TEE) increases sensitivity, while cardiac CT or MRI may be used to clarify morphology or exclude tumor thrombus [1,7]. Timely recognition is critical, but therapeutic strategies remain non-standardized and must be tailored to thrombus morphology, stability, and bleeding risk [8,9].

## Case presentation

A 49-year-old female with a history of hysterectomy and chronic right ureteral stent placement 25 years prior presented with four months of progressively worsening lower abdominal pain, constipation, dysuria, and intermittent nausea and vomiting. She had a recent hospitalization for a urinary tract infection, suspected to be related to a chronically retained and encrusted ureteral stent. Imaging at that time revealed a large, heterogeneous pelvic mass involving the bladder, rectum, anterior abdominal wall, and vaginal cuff, along with bilateral hydronephrosis. Cystoscopy with biopsy confirmed poorly differentiated urothelial carcinoma, staged T4N3M0. The patient was initiated on dose-dense MVAC (methotrexate, vinblastine, doxorubicin, and cisplatin) chemotherapy with growth factor support.

**Figure 1:**
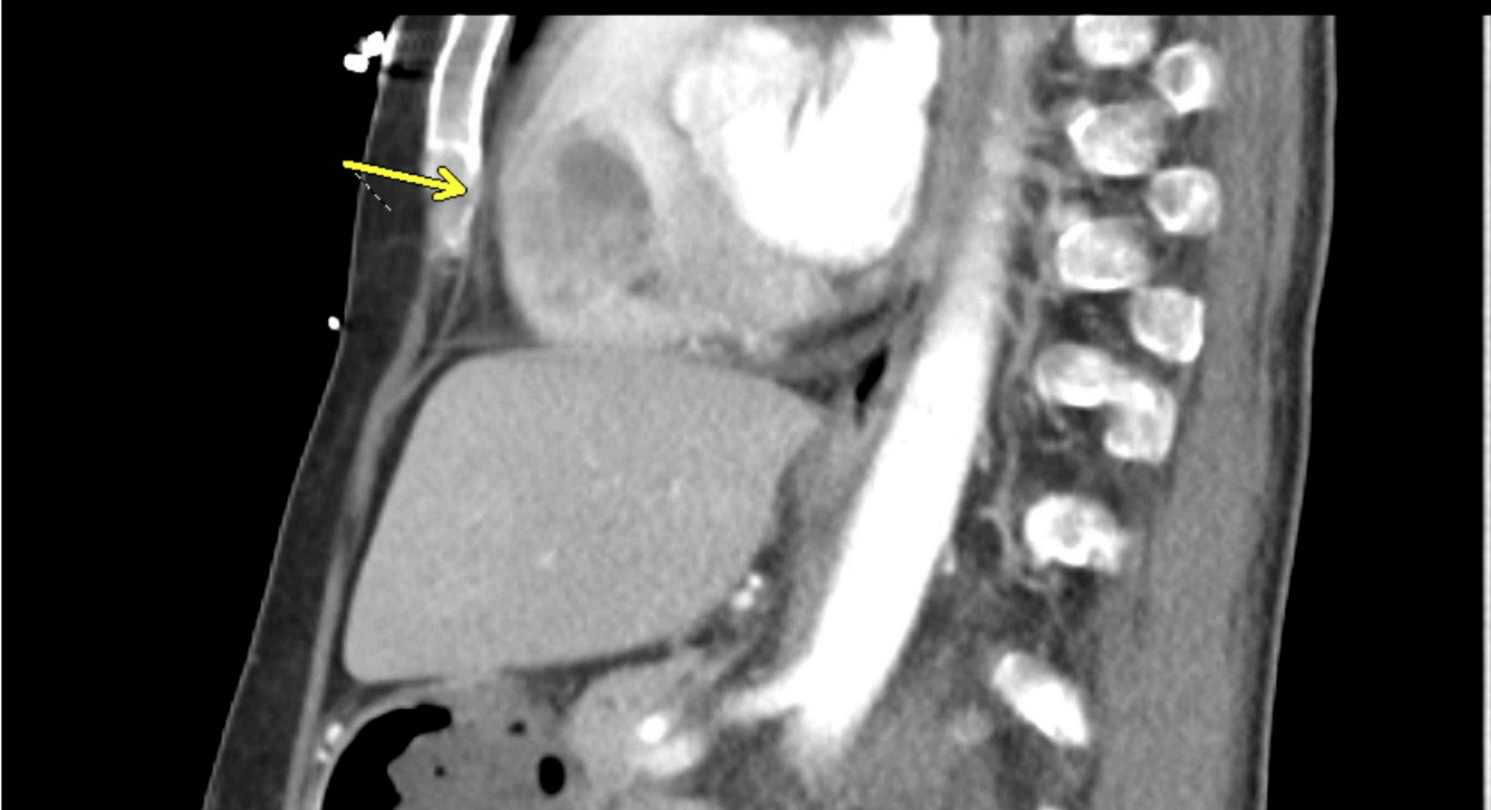
Initial sagittal post-contrast CT chest showing right ventricular filling defect (arrow).

**Figure 2:**
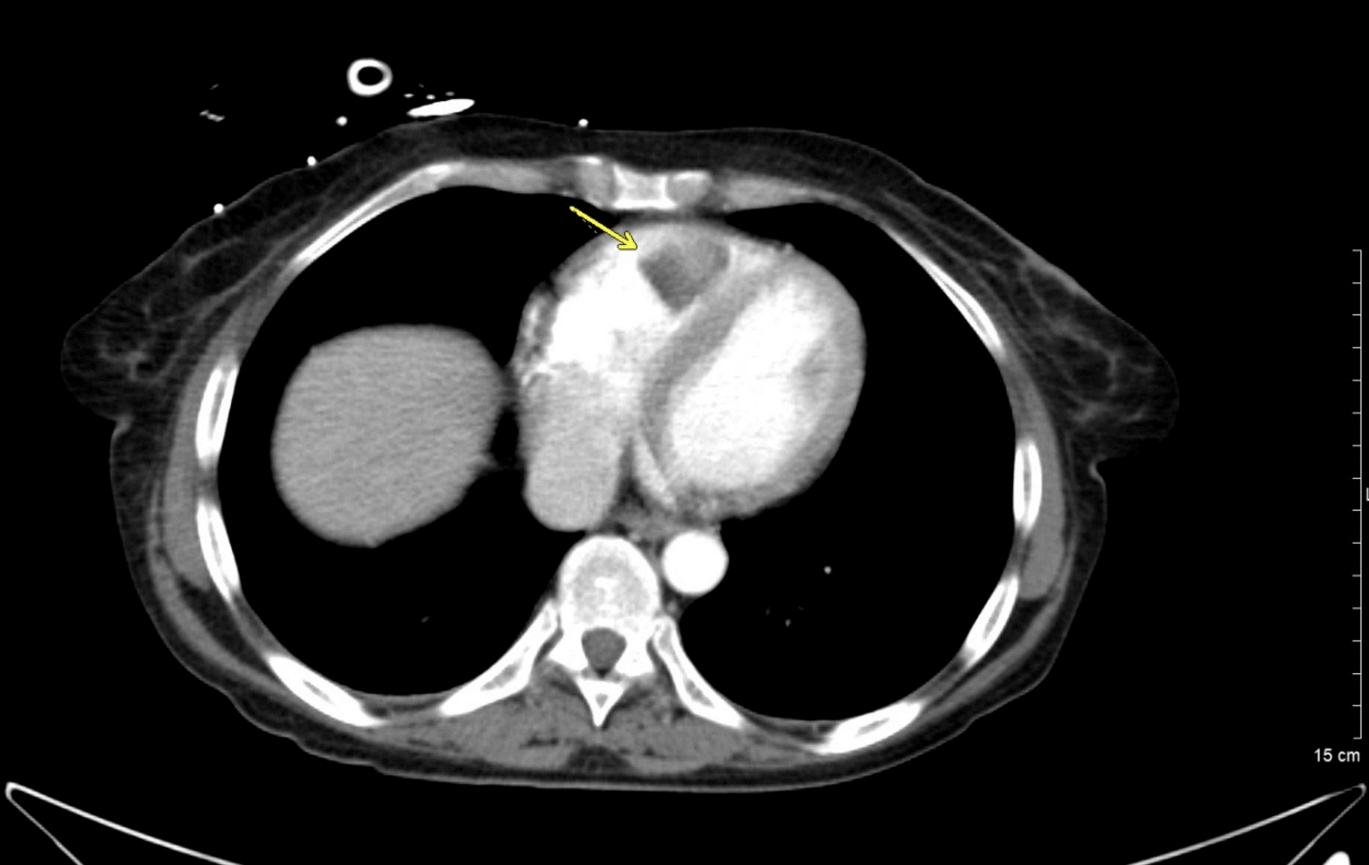
Initial axial post-contrast CT of the chest showing right ventricular filling defect (arrow).

On hospital day one, the patient developed tachycardia and hypoxia, prompting CT pulmonary angiography to evaluate for pulmonary embolism. Although the study was nondiagnostic for embolism due to motion artifact, it demonstrated a new 5-mm pulmonary nodule and a persistent filling defect in the right ventricle, raising suspicion for thrombus. Axial and sagittal CT images confirmed a right ventricular filling defect (Figures 1, 2). A TTE was subsequently obtained due to worsening oxygenation and persistent tachycardia, revealing a 3.0 × 2.2 cm mobile echogenic mass in the right ventricular outflow tract (RVOT). The right ventricle was mildly dilated with moderate systolic dysfunction, and the left ventricular ejection fraction was preserved at 60-65%. These findings were suggestive of a venous thrombus in transit versus a tumor thrombus.

The patient was initiated on intravenous heparin infusion; however, anticoagulation was discontinued shortly after due to vaginal bleeding and worsening thrombocytopenia. Baseline laboratory workup revealed a platelet count of 62,000/µL, prothrombin time (PT)/international normalized ratio (INR) of 1.4, and elevated CRP. Approximately two weeks into admission, she experienced hemodynamic collapse with supraventricular tachycardia, hypotension, and hypoxia, consistent with obstructive shock, requiring synchronized cardioversion, adenosine, vasopressor support, and transfer to the medical ICU. At this point, a disseminated intravascular coagulation (DIC) panel was collected, showing PT of 23.1 seconds, INR of 2.17, activated partial thromboplastin time (aPTT) of 38.6 seconds, fibrinogen of 155 mg/dL, and a markedly elevated D-dimer level at >10,000 ng/mL, consistent with a consumptive coagulopathy.

Repeat limited TTE confirmed persistence of the RVOT thrombus, largely unchanged in size and mobility. Given her instability and contraindications to surgery, interventional radiology attempted catheter-based thrombectomy using the AngioVac system (AngioDynamics Inc., Latham, NY), but the procedure was unsuccessful. Pathology from aspiration material revealed no malignant cells, supporting the diagnosis of bland thrombus.

**Figure 3:**
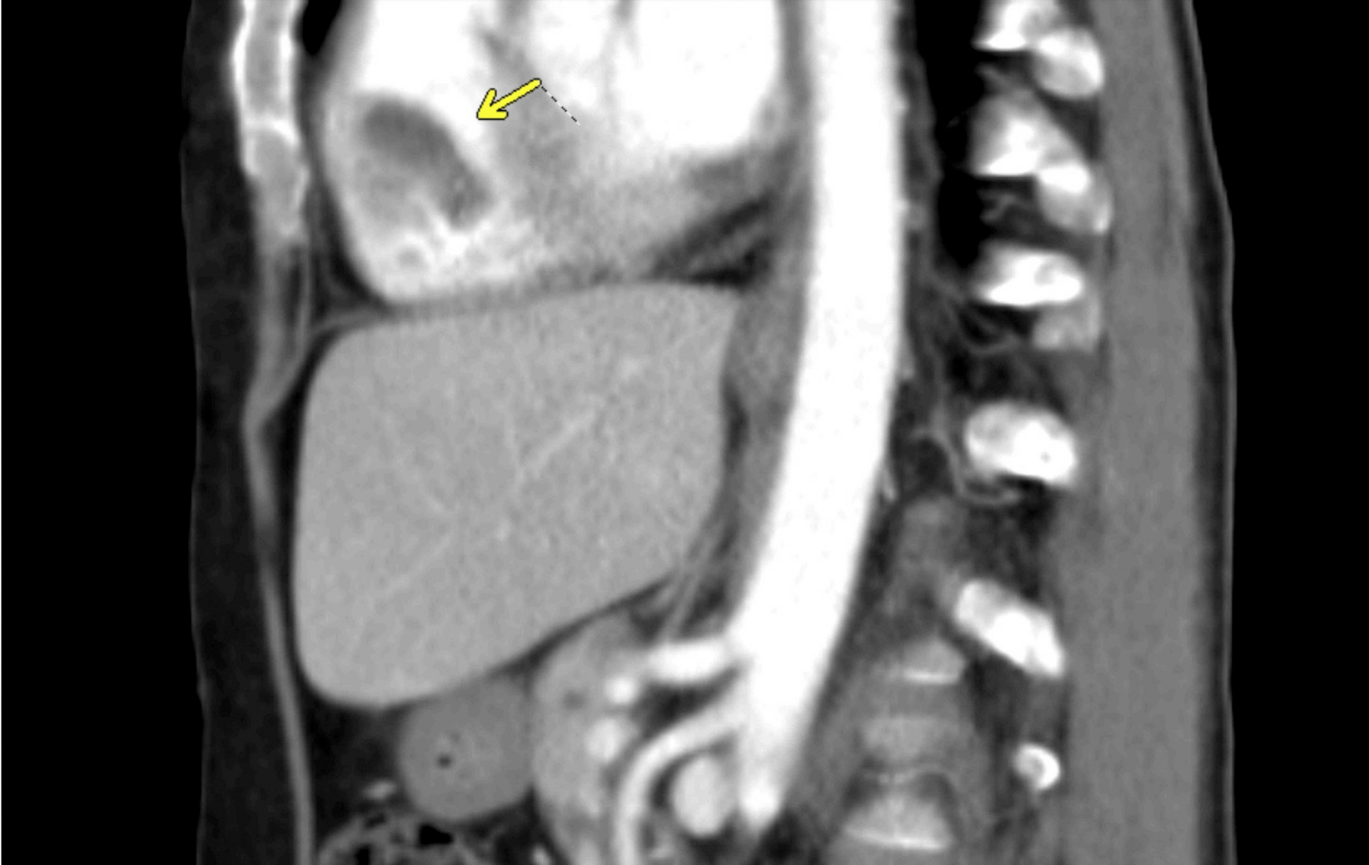
Repeat sagittal post-contrast CT of the chest showing persistent right ventricular thrombus (arrow), unchanged in size and morphology.

**Figure 4:**
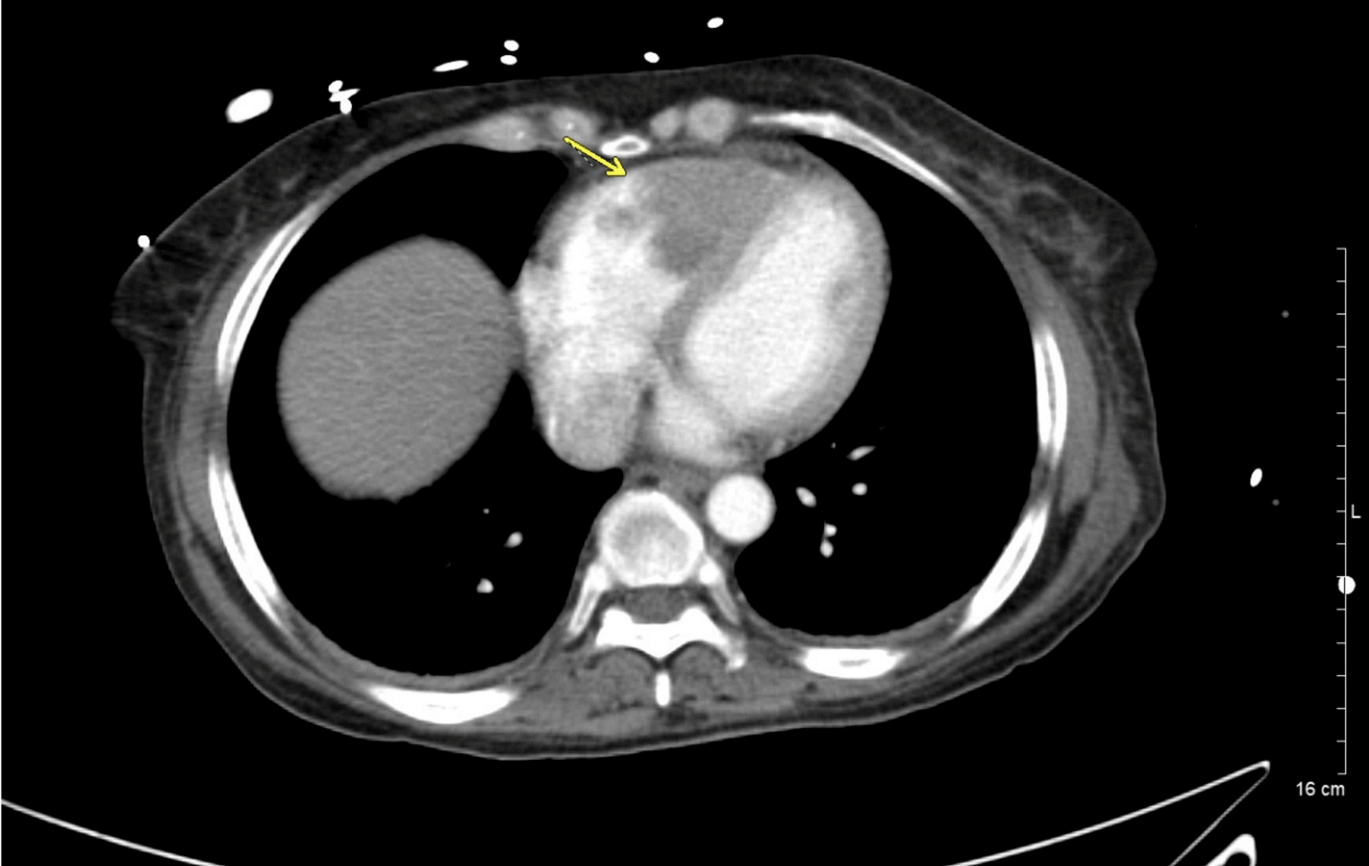
Repeat axial post-contrast CT of the chest showing persistent right ventricular thrombus (arrow), unchanged in size and morphology.

**Figure 5:**
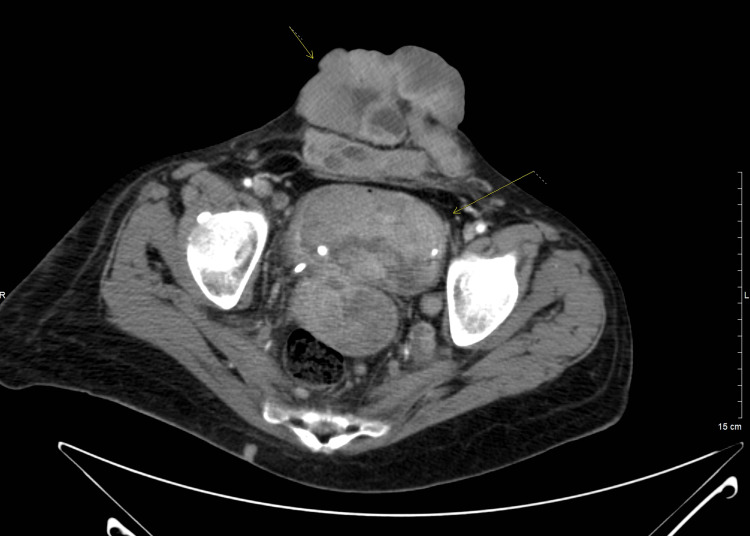
Contrast-enhanced CT of the abdomen and pelvis (axial view) demonstrating a large bladder mass (bottom arrow) involving the bladder with peritoneal metastasis (top arrow).

A follow-up CT of the chest several days later again demonstrated the persistent RVOT thrombus, unchanged in size and morphology. The sagittal view highlights the intraluminal filling defect within the RVOT (Figure 3), while the axial view confirms the same finding (Figure 4). On lung-window images (not shown), new subpleural pulmonary nodules were noted, concerning for metastasis. Concurrent CT of the abdomen/pelvis revealed interval growth of the bladder mass with stable lymphadenopathy (Figure 5).

Throughout her hospitalization, serial echocardiograms continued to demonstrate persistence of the RVOT thrombus. Additional vascular imaging revealed extensive lower-extremity deep vein thromboses, including complete occlusion of the left iliac and femoral veins. She subsequently developed methicillin-resistant *Staphylococcus aureus* (MRSA) bacteremia, progressive anemia requiring transfusions, and acute kidney injury, further complicating management. An inferior vena cava (IVC) filter was placed as anticoagulation remained contraindicated. Despite aggressive medical management, including four cycles of chemotherapy, the patient’s condition worsened with refractory hypotension, worsening organ dysfunction, and respiratory failure, requiring intubation. Following goals-of-care discussions, she was transitioned to comfort measures and ultimately passed away following compassionate extubation.

## Discussion

RVT, particularly when mobile and located in the RVOT, carries a high risk of embolization and mortality [1,6]. Differentiating thrombus in transit from tumor thrombus or vegetation requires multimodal imaging, with echocardiography as the diagnostic cornerstone [4,6]. In our patient, initial suspicion of tumor thrombus was excluded by aspiration pathology, confirming bland thrombus.

Management remains controversial. Options include anticoagulation, thrombolysis, surgical embolectomy, or percutaneous thrombectomy [2,3,8]. Some series report improved survival with thrombolysis compared to anticoagulation alone [2,5,10,11]. However, in this case, anticoagulation and thrombolysis were contraindicated by active bleeding, thrombocytopenia, and coagulopathy, while surgical embolectomy was precluded by instability and comorbidities. AngioVac thrombectomy was attempted but failed due to thrombus adherence, underscoring technical limitations.

Her clinical deterioration was multifactorial: RVOT obstruction, extensive deep vein thrombosis (DVT), sepsis, and progression of malignancy. Infection (MRSA bacteremia) and chemotherapy-associated cytopenias further restricted therapeutic options. Prognosis is especially poor in malignancy-associated RVT, where outcomes are limited by both tumor burden and bleeding risk [6,12].

This case emphasizes the importance of multidisciplinary input, including cardiology, oncology, interventional radiology, and palliative care, to balance aggressive measures with patient-centered outcomes. No consensus guidelines currently exist for RVT management in oncology patients. Research is needed to clarify best practices and to define the role of catheter-based therapies, systemic thrombolysis, and early palliative integration in this high-risk group.

## Conclusions

RVT is rare, often fatal, and especially challenging in oncology patients with contraindications to anticoagulation or thrombolysis. This case illustrates the diagnostic uncertainty and therapeutic limitations of RVT in the setting of advanced urothelial carcinoma, bleeding, and sepsis. The poor outcome despite multidisciplinary efforts highlights the urgent need for consensus guidance and research in cancer-associated RVT. Early multidisciplinary involvement and proactive palliative discussions are essential to align treatment with patient values and optimize quality of life when curative interventions are not feasible.
